# Study on Aroma Formation During the Withering Period of Ningchow Black Tea

**DOI:** 10.3390/foods15111903

**Published:** 2026-05-28

**Authors:** Yingjie Huang, Ziyi Li, Yumei Ke, Juan Tu, Feng Xie, Caigang Yan, Kai Zhong, Qincao Chen

**Affiliations:** 1 College of Agriculture, Jiangxi Agricultural University, No. 1101 Zhimin Avenue, Nanchang 330045, China; hyj605791@163.com (Y.H.); 18931077210@163.com (Z.L.); 13396161836@163.com (Y.K.); 2 Horticulture Research Institute, Jiangxi Academy of Agricultural Sciences, No. 602 Nanlian Avenue, Nanchang 330200, China; tomato.t@163.com (J.T.); xfeng415@163.com (F.X.); 3 Tonggu Agriculture, Rural Affairs and Water Resources Bureau, Yichun 336299, China; yancaigang@126.com (C.Y.); kk17891023533@163.com (K.Z.)

**Keywords:** Ningchow black tea, aroma, tea withering, volatile compound, enzyme

## Abstract

Withering is the first process in black tea production, during which volatile compounds undergo complex enzymatic transformations that significantly influence the aroma profile of the tea. Herein, this study investigated the change trends of volatile compounds and corresponding enzymes throughout the withering period of Ningchow black tea. Sensory evaluation showed that withering enhanced the aroma quality of the tea leaves. A total of 165 volatile compounds were identified using gas chromatography–mass spectrometry. After withering, the flavor index displayed a significant decrease. Among the 100 differential volatile compounds (*p* < 0.01), the top up-regulated volatiles were primarily fatty acid-derived volatiles, while the top down-regulated volatiles originated from diverse precursors. The contents of most fatty acid-, amino acid-, and carotenoid-derived alcohols and aldehydes were significantly increased, while most volatile terpenoids and other volatiles showed opposite change trends. Consistent with these changes, the activities and protein levels of key enzymes largely paralleled the alterations in volatile compounds. This study provides a detailed characterization of the formation of volatile components during the withering stage of Ningchow black tea production.

## 1. Introduction

Tea, as one of three major non-alcoholic beverages globally, is renowned for its flavor and health benefits and thus has always been deeply loved by consumers. Owing to the fermentation degree and flavor difference, teas are classified into green, yellow, white, oolong, black, and dark teas [1]. Congou black tea is the main type of black tea widely produced in China among three categories of black tea. In China, famous congou black teas include Ningchow black tea, Keemun black tea, Yingde black tea, Dian black tea, and Tanyang black tea. Among them, Ningchow black tea is the earliest congou black tea appearing in China, and is produced in Xiushui, Tonggu, and Wuning counties of Jiangxi Province [2].

Aroma is a very important factor determining tea quality, and it accounts for a proportion of 25% in sensory evaluation in China. Ningchow black tea is renowned for its strong and long-lasting sweet aroma [2,3]. Our previous study showed that the volatile components of Ningchow black tea mainly consist of esters and alcohols, and methyl salicylate, linalool, geraniol, β-ionone, phytol, and phenylacetaldehyde are the prominent aroma components [2]. Odor activity value (OAV) showed that geraniol, methyl salicylate, phenethyl alcohol, *trans*-linalool oxide (pyranoid), benzene acetaldehyde, β-myrcene, and *E*-nerolidol were the key odor-presenting substances for Ningchow black tea [3].

The compositions and contents of aroma components in teas are determined simultaneously by the fresh leaves and the processing procedures [4]. The processing procedures of black tea include withering, rolling, fermentation, first drying, and final drying [5]. Although the main purpose of withering is to evaporate appropriate moisture, which serves as a foundation for the subsequent rolling and fermentation processes, it also has profound effects on the physiological and chemical properties and subsequently the aroma compositions of tea leaves [1]. As the withering proceeds, tea leaves suffer from both the mechanical damage stress induced by picking and the dehydration stress [6]. These two stresses induce tea leaves to strengthen the synthesis and the release of volatile components [1,7]. In fresh tea leaves, volatile components can be divided into fatty acid-derived volatiles (FADVs), amino acid-derived volatiles (AADVs), volatile terpenoids (VTs), and carotenoid-derived volatiles (CDVs) based on their biosynthesis pathways and precursors [4,8]. Lipoxygenase (LOX) [9], alcohol dehydrogenase (ADH) [9], phenylalanine ammonia-lyase (PAL) [10], terpene synthase (TPS) [11], and carotenoid cleavage dioxygenase (CCD) [12,13] are directly involved in the synthesis of aroma compounds. However, the internal connection between aroma components and these enzymes during the withering step of Ningchow black tea is unclear up to now.

The aims of this study were to systematically investigate the changes in volatile compounds during the withering period of Ningchow black tea via headspace solid-phase microextraction–gas chromatography–mass spectrometry (HS-SPME-GC-MS), and to clarify the roles of related key enzymes during this period. This study will provide the first discovery of the association between a certain type of enzymes and specific aroma components during the withering period of Ningchow black tea.

## 2. Materials and Methods

### 2.1. Chemicals and Reagents

Deionized water was produced using a Milli-Q water purification system (Milli-pore, Billerica, MA, USA). *n*-Alkanes (C7–C40) were obtained from the J&K Scientific Corporation (Beijing, China). Ethyl caprate (99.0%) was obtained from the Sigma-Aldrich Corporation (Shanghai, China). The detection kits of LOX (LE-1-141), ADH (LE-1-140), and β-glucosidase (β-GD) (LE-1-108) were purchased from Hefei Lai Er biotechnology Co., LTD (Heifei, China). The detection kits of PAL (M0110A), TPS (MX-77242B2), and CCD (MX-77243B2) were purchased from Suzhou Michy Biomedical Technology Co., Ltd. (Suzhou, China).

### 2.2. Tea Samples

The clonal tea variety of Ningzhou No. 2 (*Camellia sinensis* (L.) O. Kuntze) was selected for this study. Tea trees were planted in the tea garden of Jiangxi Tonggu Tea Co., Ltd. (Yichun, China). The withering experiment commenced around 12:00 noon on 15 April 2024, and lasted for approximately 20 h. The picking standard of fresh leaves was one bud with two leaves, totaling approximately 20 kg. After transporting to the processing factory, fresh tea leaves were evenly spread out in the withering trough for natural withering without forced air or heating, and the thickness of tea leaves was about 1 cm. The temperature and humidity were in the ranges of 22.0–30.0 °C and 71–86%, respectively. According to the moisture of tea leaves during withering (Table S1), fresh tea leaves (F) and withered tea leaves for 12 h (W12) and 20 h (W20) were included as samples and were immediately frozen in liquid nitrogen. The samples were processed for one biological replicate and collected using the five-point sampling method to reduce errors. A part of the samples was vacuum freeze-dried (LC-10N-80A; Lichen, Changsha, China) at −55 °C for 36 h and stored at −20 °C for sensory evaluation and GC-MS analysis. The remaining samples were stored at −80 °C for the determination of enzyme activity.

### 2.3. Sensory Evaluation of Tea Samples

Sensory evaluation was carried out according to the Chinese national standard of “Methodology of sensory evaluation of tea” (GB/T 23376–2018) and “Tea vocabulary for sensory evaluation” (GB/T 14487–2017). Non-powdered tea samples (3.0 g) were brewed with 150 mL of boiled water in a specialized cup for 5.0 min. After brewing, the tea infusions were transferred to a specialized bowl, and then the aroma of the samples was evaluated by three experienced assessors.

### 2.4. GC-MS Analysis of Volatile Compounds

The analysis of volatile compounds was performed using HS-SPME-GC-MS according to the method described in our previous study [14]. In line with the Chinese national standard method (GB/T 8303-2013, Tea—Preparation of ground sample and determination of dry matter content), tea samples were thoroughly ground with a mortar and then sieved through a 1000 μm sieve. Powdered tea samples (1.0 g) were first placed in a 20 mL glass vial (O.D. × H 22.5 × 75.5 mm; Supelco, Bellefonte, PA, USA), followed by the addition of 16 µL of ethyl caprate solvent (dissolved in ethanol, 100 mg/L) as an internal standard (IS). Subsequently, 8 mL of boiling deionized water was added into the vial, which was immediately sealed with a cap and heated at 60 °C for 3.0 min using a heating oscillator. Volatile compounds were adsorbed for 60 min with a Carboxen/polydimethylsiloxane (CAR/PDMS)-coated fiber (85 µm, 1 cm; Supelco, Inc., Bellefonte, PA, USA). Finally, the volatile compounds were desorbed at 250 °C for 5 min for GC–MS analysis.

GC–MS analysis was conducted on a GC system (8860, Agilent Technologies, Santa Clara, CA, USA) coupled with an MS detector (5977B, Agilent Technologies, Santa Clara, CA, USA), using a HP-5MS column (30 m × 250 µm × 0.25 µm, Agilent Technologies; Santa Clara, CA, USA). Helium (99.999%) served as the carrier gas at a constant flow rate of 1.6 mL/min. The oven temperature was programmed as follows: hold at 40 °C for 5 min, ramp to 160 °C at 3 °C/min, then to 250 °C at 15 °C/min, and hold for 5 min. Splitless injection was employed with a purge time of 6 min. MS analysis was performed under the following conditions: EI mode; ionization voltage, 1196 V; ion source temperature, 230 °C; quadrupole temperature, 150 °C; full scan range, *m*/*z* 33–600 amu; electron ionization energy, 70 eV. Each sample was technically analyzed in quadruplicate.

Raw data were processed using LECO Chroma TOF software (V 4.51.6.0). The parameters for processing individual sample data were as follows: minimum signal-to-noise ratio (S/N) of 20; minimum similarity match of 750 (maximum of 1000); and first-dimension peak width of 25. The integrated peak table for all samples was generated using the statistical comparison function within the same software, with a retention time (RT) deviation of 0.2 min. Retention indices (RIs) of volatile compounds were automatically calculated using a series of *n*-alkanes (C7–C40). Volatile compounds with an RI difference of less than 20 between the experimental and reference values were retained for further analysis. The referred RIs of volatile compounds were obtained from the NIST library (V 14.L). The concentrations of volatile compounds were calculated using the following formula: C (µg/L) = 200 × PA_V_/PA_IS_, of which PA_V_ and PA_IS_ were the peak area of volatile and internal standard, respectively.

### 2.5. Determination of the Activities and Protein Levels of Enzymes

#### 2.5.1. Determination of the Activity of LOX

The measurement method referred to the manufacturer’s instruction. At first, 3.0 g of fresh tea leaves was ground into homogeneous powders using liquid nitrogen. In accurate amounts, 0.10 g of tea powders and 1.0 mL of reagent 1 were homogenized on an ice bath. The homogenates were centrifugated at 16,000× *g* for 20 min at 4 °C, and the resulted supernatants were the crude enzyme solutions. The reaction tubes were 1 cm quartz cuvettes. The CK reaction system included 100 µL of crude enzyme solution and 900 µL of reagent 2, and the sample reaction system included 100 µL of crude enzyme solution and 900 µL of reagent 3. After reacting for 30 min at 30 °C, the absorbances of CK and sample solutions were determined at 280 nm and recorded as A1 and A2, respectively. The calculation formula was as follows: LOX (U/g) = 33.33 × (A2 − A1)/(W × (1 − C)), of which W and C were the sample weight and moisture content, respectively.

#### 2.5.2. Determination of the Activity of ADH

The measurement method referred to the manufacturer’s instruction. The preparing of crude enzyme solutions was the same to that of LOX. The reaction tubes were 1 cm quartz cuvette. The reaction system included 100 µL of crude enzyme solution, 800 µL of reagent 3, and 100 µL of reagent 4. Subsequently, the reaction solutions were thoroughly mixed and then applied to measure the absorbance at 340 nm. The absorbances at 15 s and 75 s were recorded as A1 and A2, respectively. The calculation formula was as follows: ADH (nmol/min/g) = 1608 × (A2 − A1)/(W × (1 − C)), of which W and C were the sample weight and moisture content, respectively.

#### 2.5.3. Determination of the Activity of PAL

The measurement method referred to the manufacturer’s instruction. At first, 3.0 g of fresh tea leaves were ground into homogeneous powders using liquid nitrogen. In accurate amounts, 0.10 g of tea powders and 1.0 mL of extracting solution were homogenized on an ice bath. The homogenates were centrifugated at 12,000× *g* for 10 min at 4 °C, and the resultant supernatants were the crude enzyme solutions. The reaction tubes were 1 cm quartz cuvettes. The CK reaction system included 150 µL of reagent 1 and 40 µL of reagent 2, and the sample reaction system included 5 µL of crude enzyme solution, 145 µL of reagent 1, and 40 µL of reagent 2. After reacting for 30 min at 30 °C, 10 µL of reagent 3 was added into the reaction systems. After thorough mixing and standing for 10 min, the absorbances of CK and sample solutions were determined at 290 nm and recorded as A1 and A2, respectively. The calculation formula was as follows: PAL (U/g) = 26.6 × (A2 − A1)/(W × (1 − C)), of which W and C were the sample weight and moisture content, respectively.

#### 2.5.4. Determination of the Protein Level of TPS

Enzyme-linked immunoassay (ELISA) was used to determine the protein level of TPS according to the manufacturer’s instruction. Fresh tea leaves were ground to a fine powder in liquid nitrogen. Afterwards, 1.0 g amounts of powders were placed in a centrifuge tube containing 5 mL of phosphate buffer (pH 6.8, 0.01 M). The solutions were centrifuged at 3000 rpm for 20 min at 4 °C and the resulting supernatants were collected for the ELISA assay. Samples were evaluated in a 96-well plate using a microplate reader according to the following protocols: (1) 50 µL standards of different concentrations were added to the specified locations; correspondingly, 10 µL of sample solution and 40 µL of diluent were added to the sample wells; and the blank wells were designated at specified locations and lacked the sample solution and HRP conjugate reagent; (2) with the exception of blank wells, 100 µL HRP conjugate reagents were added to each well; (3) after sealing the wells with film, the 96-well plate was incubated at 37 °C for 60 min; (4) after discarding the solutions, washing buffers were added to each well and were then discarded 1 min later; this process was repeated 5 times; (5) 50 µL of substrate A and B were added to each well and incubated for 15 min at 37 °C under dark conditions; and (6) 50 µL of stop solution was added to each well, and the absorbances were measured at 450 nm within 15 min after stopping the reaction.

#### 2.5.5. Determination of the Protein Level of CCD

The protein level of CCD was also determined using the ELISA method according to the manufacturer’s instructions, using the same operating steps and parameters as the TPS ELISA method, except that a different specific antibody was employed.

#### 2.5.6. Determination of the Activity of β-GD

The measurement method referred to the manufacturer’s instructions. At first, 3.0 g of fresh tea leaves was ground into homogeneous powders using liquid nitrogen. In accurate amounts, 0.10 g of tea powder and 1.0 mL of extracting solution were homogenized on an ice bath. The homogenates were centrifugated at 15,000× *g* for 10 min at 4 °C, and the resultant supernatants were the crude enzyme solutions. The reaction vessels were 1 cm glass colorimeters. The CK reaction system included 400 µL of distilled water, 500 µL of reagent 2, and 100 µL of crude enzyme solution, and the sample reaction system included 400 µL of reagent 1, 500 µL of reagent 2, and 100 µL of crude enzyme solution. After reacting for 30 min at 37 °C, the reaction systems were incubated at 95 °C for 5 min and were then cooled using flowing water. The cooled solutions were centrifugated at 8000× *g* for 5 min at 4 °C, and the supernatants (500 µL) were mixed with 1000 µL of reagent 3. After mixing and standing for 2 min, the absorbances of CK and sample solutions were determined at 400 nm and recorded as A1 and A2, respectively. The calculation formula was as follows: β-GD (nmol/min/g) = 61.39 × (A2 − A1 + 0.0027)/(W × (1 − C)), of which W and C were the sample weight and moisture content, respectively.

### 2.6. Data Processing Method

Principal component analysis (PCA) was performed using Simca-P 13.0 software (Umetrics AB, Umeå, Sweden) with the Pareto-scaling mode. The significance of differences in overall comparisons was assessed by Tukey s-b(K) test using PASW Statistics software (Version 18.0, Chicago, IL, USA). A heat map was generated by using MultiExperiment Viewer 4.8.1 (Oracle Corporation, Redwood, CA, USA) after UV-scaling of the data.

## 3. Results and Discussion

### 3.1. Sensory Evaluation Results of Tea Samples

Sensory evaluation results of tea samples are shown in Table 1. Fresh tea leaves (F) showed an obvious grassy odor. From F to W20, the intensity of grassy odor was gradually decreased, while the intensity of green odor showed a rising trend. For W20, it emitted a moderate sweet and/or floral odor. Our previous study also showed that withering tea leaves had a faint floral scent [14]. The above results indicate that withering is beneficial to improve the aroma quality of tea leaves.

### 3.2. The Profiles of Volatile Compounds

A total of 297 volatile compounds were firstly obtained after peak alignment. After eliminating the unreliable volatiles by the RI comparison, 165 volatile compounds were retained for subsequent analysis (Table S2). Those volatile compounds were composed of 8 aromatic hydrocarbons, 13 alkanes, 26 alkenes, 28 alcohols, 26 aldehydes, 11 ketones, 43 esters, 7 oxygen heterocyclic compounds, and 3 other compounds (Table S2). In fresh tea leaves, the ester was the predominant category, reaching 3711.33 µg/L and accounting for 27.89%, and other fragrances with high levels included the alcohols (3372.30 µg/L, 24.89%), alkenes (3060.08 µg/L, 22.72%), and aromatic hydrocarbons (2396.38 µg/L, 17.89%) (Table 2). During the withering period, the contents of aromatic hydrocarbons, alkanes, alkenes, esters, and others were significantly decreased, while the contents of aldehydes and oxygen heterocyclic compounds were markedly increased (Table 2), which is similar with the results in our previous study [4]. In terms of individual volatiles, according to their average contents, the predominant volatile compounds were linalool, styrene, (*Z*)-3-hexenyl acetate, β-myrcene, limonene, phenylethyl alcohol, methyl salicylate, butyl butanoate, geraniol, (*E*)-3-hexenyl butanoate, benzyl alcohol, *trans*-linalool oxide (furanoid), *cis*-3-hexenyl hexanoate, (*E*)-4,8-dimethylnona-1,3,7-triene, *trans*-β-ocimene, and benzaldehyde amongst others (Table S2).

### 3.3. Effects of Withering on the Flavor Index

Up to now, more than 1700 volatile compounds have been identified across 90 plant families; however, their biosynthesis originates from only a few primary metabolic pathways [15]. Based on their biosynthetic precursors, volatile compounds are divided into 4 classes, including FADVs, AADVs, VTs, and CDVs [4,8]. As shown in Table 3, the total contents ranked in descending order as VTs, FADVs, AADVs, and CDVs. From stage F to W20, the total content of FADVs showed a significant decreasing trend. From the perspective of chemical structure classification, the total content of non-ester FADVs was significantly up-regulated, whereas the ester FADVs showed an opposite trend. The total contents of AADVs and CDVs also significantly increased, while that of VTs decreased slightly but not significantly (Table 3).

**Figure 1:**
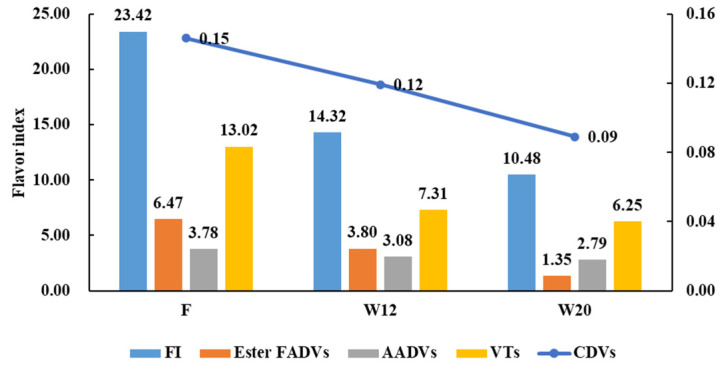
The changes in flavor index of various types of volatile compounds. F: fresh tea leaves; W12 and W20: tea leaves were withered for 12 h and 20 h, respectively.

These volatile components exhibit complex and variable odors, such as green, grassy, sweet, floral, and fruity; and thus, they are divided into two groups. Group I compounds impart an undesirable grassy and/or green odor, and non-ester FADVs, especially C6–C9 alcohols and aldehydes, make up over 90% of Group I; Group II compounds impart a sweet flowery aroma to black tea, and are mainly composed of AADVs, VTs, and CDVs [16,17]. In addition, most ester FADVs also impart pleasant fruity, floral, fresh scents, and thus were classified into the Group II [18]. The ratio of the sum of Group II compounds to that of Group I compounds is the flavor index (FI), which positively reflects the aroma quality of tea [16,17]. Some esters, such as hexyl isobutyrate, *cis*-3-hexenyl isovalerate, and cis-3-hexenyl benzoate, belong to both FADVs and AADVs. As their scents and change trends were in line with that of AADVs, they were classed into AADVs when calculating FI. As shown in Figure 1, the FI presented a downward trend. For subclasses, the individual FI of ester FADVs, AADVs, VTs, and CDVs were all largely declined (Figure 1). The decrease in FIs attributed to the significant increase in non-ester FADVs (Table 3). In terms of FI, withering seems to be not conducive to forming a more advantageous aroma composition, which is in conflict with the sensory evaluation results. However, the interaction, including synergistic, inhibiting, and additive effect, between aroma components is a common phenomenon. Yang et al. found that β-ionone masked the green odor intensity of heptanal and promote the formation of sweet floral aroma in black tea [19]. In addition, a previous study showed that the green odor of (*E*)-2-hexenal was masked by β-ionone and β-damascenone at the threshold concentrations [20]. The possible reason is that the sweet and/or floral substances inhibit and/or alter the fragrance of grassy substances.

### 3.4. Principal Component Analysis of Volatile Compounds

**Figure 2:**
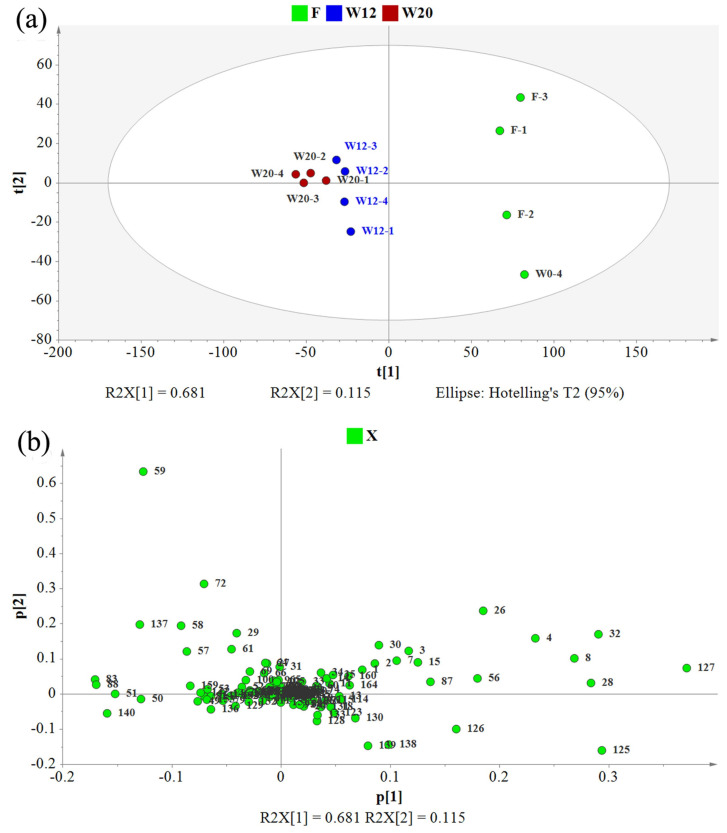
Principal component analysis of volatile compounds of tea samples. (**a**) The score plot, R^2^X = 0.797, Q^2^ = 0.519; (**b**) the loading plot, and the compounds replaced by numbers correspond to the substances with the same numbers in Table S2. The data were Pareto scaled. F: fresh tea leaves; W12 and W20: tea leaves were withered for 12 h and 20 h, respectively.

After pretreatment of the data of volatile components, an unsupervised PCA approach was performed to obtain an overview of the differences in volatile components among tea samples. As shown in Figure 2a, three groups were clearly separated in the PCA score plot, indicating distinct metabolome patterns among them. The first and second principal components explained 68.1% and 11.5% of total variance, respectively. Additionally, a PCA-loading plot was applied to investigate the primary differential volatile compounds. As shown in Figure 2b, the noticeable contributors to the differences among tea samples included (*Z*)-3-hexenyl acetate (127), butyl butanoate (125), (*E*)-4,8-dimethylnona-1,3,7-triene (32), limonene (28), naphthalene (8), linalool (59), styrene (4), β-myrcene (26), benzyl alcohol (56), ethyl hexanoate (126), (*E*,*E*)-2,4-heptadienal (87), benzaldehyde (83), and benzene acetaldehyde (88).

### 3.5. Effects of Withering on Individual Volatile Components

#### 3.5.1. Top Differential Volatile Compounds During the Withering Period

**Figure 3:**
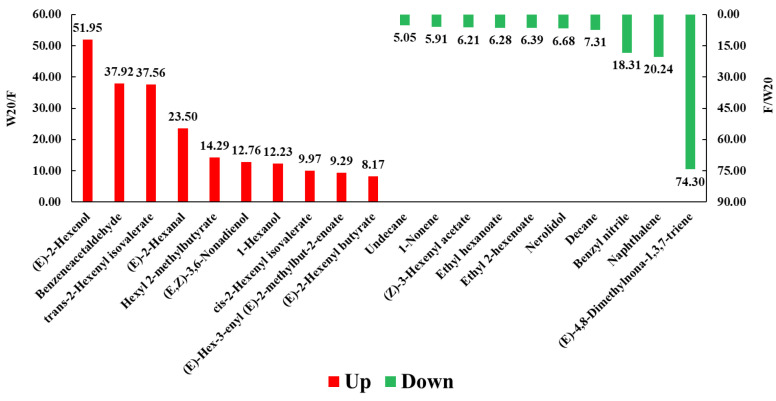
Fold changes of the top changed volatile compounds. The numbers represent the increasing (Up) or decreasing (Down) fold. F: fresh tea leaves; W20: tea leaves withered for 20 h.

The differential volatile components among samples were screened via the ANOVA (*p* < 0.01). In total, 100 volatile compounds showed significant difference; of which, the concentrations of 52 volatile substances were largely elevated, while those of 48 volatile compounds were largely decreased after withering (W20/F). The top 10 increasing volatile compounds were (*E*)-2-hexenol, benzeneacetaldehyde, *trans*-2-hexenyl isovalerate, (*E*)-2-hexanal, hexyl 2-methylbutyrate, (*E*,*Z*)-3,6-nonadienol, 1-hexanol, *cis*-2-hexenyl isovalerate, (*E*)-hex-3-enyl (*E*)-2-methylbut-2-enoate, and (*E*)-2-hexenyl butyrate, and they were almost all FADVs (Figure 3). It implied that the metabolic pathway of fatty acids responded most strongly to the stress during the withering process compared with the other three synthesis pathways. The top 10 decreasing volatile compounds were (*E*)-4,8-dimethylnona-1,3,7-triene, naphthalene, benzyl nitrile, decane, nerolidol, ethyl 2-hexenoate, ethyl hexanoate, (*Z*)-3-hexenyl acetate, 1-nonene, and undecane (Figure 3), and they originate from diverse precursors. In comparison, the increase factors were greater than the decrease factors for the top differential volatiles. This was in line with the greatest increase in the content of FADVs (Table 2), and also provided a clear illustration of the phenomenon that although the contents of other volatiles have increased, the flavor index still decreased (Figure 1).

#### 3.5.2. Changes in Differential Volatile Compounds During the Withering Period

**Figure 4:**
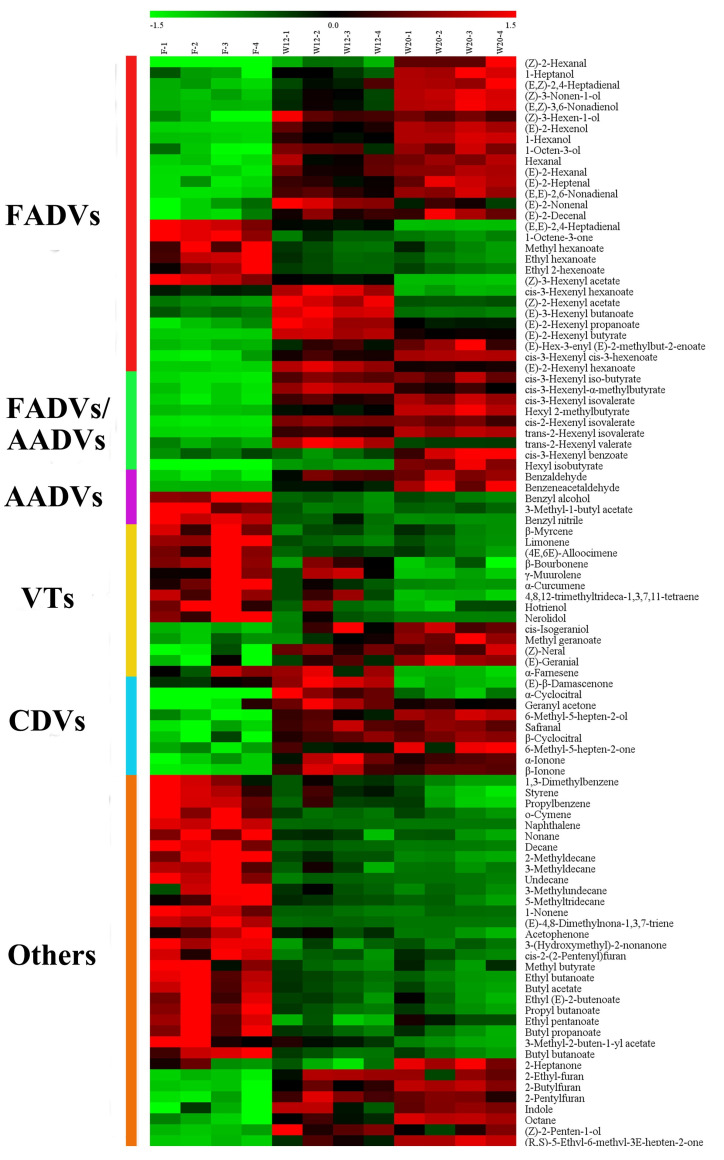
Heat map of the contents of significantly changed volatile compounds (ANOVA, *p* < 0.01). The data were UV scaled. F: fresh tea leaves; W12 and W20: tea leaves were withered for 12 h and 20 h, respectively.

According to their biosynthetic pathways, the differential volatile components included 66 FADVs, AADVs, CDVs, and VTs, and 34 other compounds (Figure 4).

FADVs are important secondary volatile metabolites in tea plants and play a significant role in contributing to tea aroma. Specifically, unsaturated fatty acids are first converted into volatile aldehydes through the action of LOX and hydroperoxide lyase; subsequently, volatile aldehydes are sequentially converted into volatile alcohols and esters by ADH and alcohol acyltransferase [21]. In addition, methyl ketones are derived from β-oxidative degradation of fatty acids [22]. As shown in Figure 4, the levels of most alcohol- and aldehyde-type FADVs, along with certain ester-type, including (*Z*)-2-hexanal, 1-heptanol, (*E*,*Z*)-2,4-heptadienal, (*Z*)-3-nonen-1-ol, (*E*,*Z*)-3,6-nonadienol, hexanal, *cis*-3-hexenyl *cis*-3-hexenoate, (*E*)-2-hexenyl hexanoate, and *cis*-3-hexenyl iso-butyrate, were significantly improved. In contrast, the levels of (*E*,*E*)-2,4-heptadienal, 1-octene-3-one, methyl hexanoate, ethyl hexanoate, ethyl 2-hexenoate, and (*Z*)-3-hexenyl acetate were significantly down-regulated. Moreover, *cis*-3-hexenyl hexanoate, (*Z*)-2-hexenyl acetate, (*E*)-3-hexenyl butanoate, (*E*)-2-hexenyl propanoate, (*E*)-2-hexenyl butyrate, and *cis*-3-hexenyl-α-methylbutyrate exhibited a trend of first increasing and then decreasing (Figure 4). Overall, most FADVs showed an enhanced biosynthetic tendency during the withering period, which is similar with that of white tea withering [4]. The content enhancement of FADVs is attributed the fact that under dehydration stress, tea plants synthesize and release a series of aroma compounds as signaling molecules into the air [6,7].

AADVs are composed of volatile phenylpropanoid/benzenoids (VPBs) derived from phenylalanine, as well as volatile compounds derived from branched-chain amino acids and methionine [15]. VPBs include benzaldehyde, benzeneacetaldehyde, benzyl alcohol, phenyl ethanol, benzyl nitrile, and methyl salicylate [15]. Branched-chain amino acids, namely leucine, *iso*-leucine, and valine, can be synthesized to 3-methyl-butanal, 2-methyl-butanal, and 2-methyl-propanal, along with their corresponding alcohols and esters [15,22]. Methionine can be synthesized to methanethiol, dimethyl sulfide, and dimethyl disulfide [22,23]. As shown in Figure 4, the significantly increased AADVs were mainly esters and aldehydes, including *cis*-3-hexenyl *iso*-butyrate, *cis*-3-hexenyl isovalerate, hexyl 2-methylbutyrate, *cis*-2-hexenyl isovalerate, *trans*-2-hexenyl isovalerate, *cis*-3-hexenyl benzoate, hexyl isobutyrate, benzaldehyde, and benzeneacetaldehyde. In contrast, the significantly decreased AADVs were benzyl alcohol, 3-methyl-1-butyl acetate, and benzyl nitrile. In addition, the contents of methyl salicylate and dimethyl sulfide were also significantly increased (*p* < 0.05, Table S2). The increasing of most AADVs was also due to the dehydration stress similar with FADVs. It was shown that the drought stress induced tea plants to strengthen the synthesis of volatile phenylpropanoid/benzenoids [24]. Actually, the dehydration stress will cause the plants to suffer from water shortage, which is similar with the drought stress. In addition, it has shown that the content of phenylalanine increases significantly during the withering process [4], which will provide a rich precursor for synthesizing VPBs.

VTs is the most diverse group of volatile organic compounds comprising the C5 isoprene, C10 monoterpenes, C15 sesquiterpenes, C11 and C16 homoterpenes, and some C20 diterpenes [25]. In the present study, 38 VTs were detected and 13 VTs showed significant difference (Figure 4). Differential VTs displayed two change trends: β-myrcene, limonene, (4*E*,6*E*)-alloocimene, β-bourbonene, γ-muurolene, α-curcumene, 4,8,12-trimethyltrideca-1,3,7,11-tetraene, hotrienol, and nerolidol were significantly down-regulated; and *cis*-isogeraniol, methyl geranoate, (*Z*)-neral, and (*E*)-geranial were significantly up-regulated. Like FADVs and AADVs, it has become evident that various VTs also act as plant-to-plant signaling cues under biotic and abiotic stress [7,25]. However, recent studies have shown that TPS genes strongly responded to continuous mechanical damage, low-temperature stress, and E. (M.) onukii attack other than the dehydration stress [26,27,28]. Other studies also showed that most differential TPS genes were significantly down-regulated during the withering period [4,29]. These studies implied that the insensitivity of TPS genes to the dehydration stress was responsible for the decrease in the contents of most VTs during the withering period.

CDVs originate from the enzymatic oxidative, co-oxidation, photo-oxidation, or thermal degradation of carotenoids, such as β-carotene, α-carotene, phytoene, and lycopene, during tea processing [23,30]. In this study, 15 CDVs were detected, 10 of which showed significant differences (Table S2). As shown in Figure 4, the contents of most CDVs including geranyl acetone, 6-methyl-5-hepten-2-ol, safranal, β-cyclocitral, 6-methyl-5-hepten-2-one, α-ionone, and β-ionone showed an upward trend, which is consistent with a previous study [31]. The contents of α-farnesene, (*E*)-β-damascenone, and α-cyclocitral were raised first and then decreased (Figure 4). CDVs typically emit pleasant floral, fruity, sweet, cream-like, or woody odors [23,32]. Moreover, benefiting from their markedly low-odor threshold, several CDVs including β-ionone, α-ionone, β-damascenone, dihydroactinidiolide, and geranylacetone, have been identified as the key odorants in black teas [32,33,34]. The significant increasing in their concentrations is beneficial to improving black tea aroma.

In addition, 34 other compounds showed significant difference during the withering period (Figure 4). These differential compounds presented two opposite change trends. The decreasing compounds included 1,3-dimethylbenzene, styrene, naphthalene, nonane, decane, 1-nonene, acetophenone, methyl butyrate, ethyl butanoate, ethyl pentanoate, and butyl butanoate. The increasing compounds included 2-heptanone, 2-ethyl-furan, 2-butylfuran, 2-pentylfuran, indole, octane, (*Z*)-2-penten-1-ol, and (*R*,*S*)-5-ethyl-6-methyl-3*E*-hepten-2-one. Naphthalene is an odorant with unpleasant or pungent odors and has been identified as a key aroma-active compound in black tea [35]. 2-Pentylfuran has been shown to contribute to the chestnut-like aroma in green teas [36]. Indole is an important aroma-active compound in black tea [32]; however, it possesses a dual-odor characteristic. It emits a floral scent at an extremely low concentration; conversely, a fecal and/or animal-like odor is typically detected at higher concentrations [37].

### 3.6. Effects of Withering on the Activities of Related Enzymes

Currently, a series of enzymes involved in the biosynthesis of volatile components in tea plants have been clarified. The key enzymes involved in the biosynthesis of FADVs, AADVs, VTs, and CDVs include LOX and ADH [9], PAL [10], TPS [11], and CCD [12,13], respectively. In addition, the alcoholic volatile compounds in plants also occur in the form of glycosides, and β-GD is the key enzyme responsible for the hydrolysis of glycosidically bound volatiles (GBVs) [38]. To further clarify the roles of these enzymes in the changes of volatile compounds during the withering period, their activities were determined.

**Figure 5:**
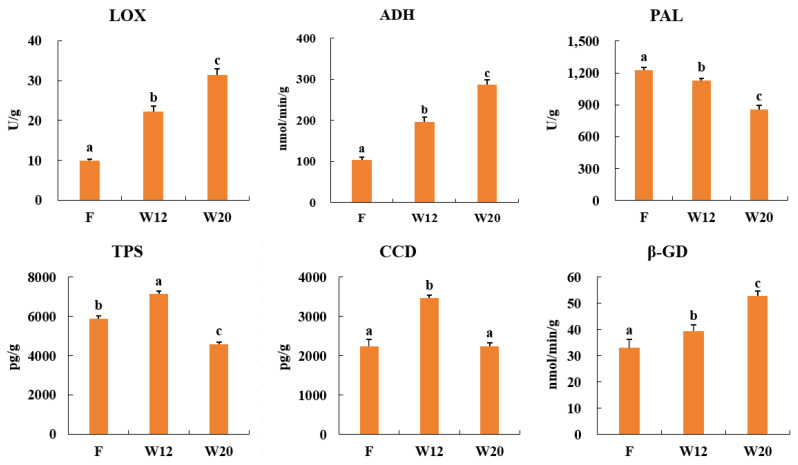
The changes in the activities and protein levels of related enzymes. LOX: lipoxygenase; ADH: alcohol dehydrogenase; PAL: phenylalanine ammonia-lyase; TPS: terpene synthase; CCD: carotenoid cleavage dioxygenase; β-GD: β-glucosidase. a, b, c: *p* < 0.05 for the changes with different letters (Tukey s-b (K) test). F: fresh tea leaves; W12 and W20: tea leaves were withered for 12 h and 20 h, respectively.

As shown in Figure 5, the activities of LOX and ADH were significantly increased, which is consistent with the previous results [39]. Contrary to LOX and ADH, the activity of PAL was significantly decreased (Figure 5), which conflicted with the change trends of main VPBs including benzaldehyde, benzeneacetaldehyde, and methyl salicylate (Figure 4). It was reported that there were three potential synthesis pathways of VPBs from phenylalanine in plants [10,40]. In addition, it was shown that the expression of phenylacetaldehyde reductase (*PAR*) and aminotransferase (*AT*) genes, the two key genes located in the other two pathways, were significantly up-regulated during the withering period [4]. Therefore, it is speculated that withering is via enhancing the *PAR* and/or *AT* located pathways to improve the amounts of VPBs. As shown in Figure 5, the protein level of TPS was firstly up-regulated within 12 h, but then was significantly down-regulated in the subsequent period (Figure 5), which was not in line with the change trends in most VTs (Figure 4). The *CCD* genes are strongly induced by abiotic stresses including dehydration stress, low-temperature stress, and mechanical damage stress [12,13]. The protein level of CCD was significantly up-regulated at 12 h (Figure 5), which is basically in line with the change trends of CDVs. CDVs can also be produced by the co-oxidation of lipid oxidative degradation [30]. The production of FADVs is a process of lipid oxidative degradation. Thus, the production of FADVs may accelerate the generation of CDVs during the withering period. In addition, the activity of β-GD was significantly raised (Figure 5). As β-GD and GBVs are separated in various cell regions, β-GD and GBVs are thus unable to directly contact with each other during the withering period [41]. Studies have shown that GBVs increase obviously during the withering process, but decrease markedly during the rolling and fermentation processes [14,42]. This implies that β-GD, although a key enzyme responsible for the hydrolysis of GBVs, is more effective during the rolling and fermentation processes. However, prolonged withering leads to gradual cell disintegration, which creates the conditions necessary for β-GD to exert its function. From this perspective, the increasing in β-GD activity will influence the accumulation of alcohol volatile compounds during the withering process. Overall, the changes in the activities of LOX, ADH, and β-GD, as well as the protein level of CCD, were well aligned with the accumulation patterns of FADVs and CDVs. In contrast, the changes in PAL activity and TPS level were not in line with that of VPBs and VTs, indicating that VPBs and VTs are subject to more complex regulatory mechanisms during the withering period.

## 4. Conclusions

In this study, the sensory characteristics and the changes in volatile compounds during the withering period of Ningchow black tea were investigated using HS-SPME-GC/MS. Sensory evaluation indicated that the aroma quality of tea leaves was improved after withering. A total of 165 volatile compounds were identified; among which the contents of aromatic hydrocarbons, alkanes, alkenes, esters, and other volatiles were significantly down-regulated, while the alcohols, aldehydes, and oxygen heterocyclic compounds exhibited reverse change trends. Owing to the substantial increasing in non-ester FADVs, the FI decreased considerably during the withering period. Among the 100 differential volatile compounds, the top increasing volatiles were dominantly occupied by FADVs. Although most of FADVs, AADVs, and CDVs were dramatically synthesized inducing by the dehydration stress, most VTs and other compounds showed contrary change trends. The changes in the LOX, ADH, CCD, and β-GD activities well supported that of FADVs and CDVs, while the changes in the PAL activity and TPS protein level were not consistent with that of VPBs and VTs, implying that VPBs and VTs are regulated by the other key enzymes during the withering period. This study provided novel insights into the aroma formation mechanism during the withering period of Ningchow black tea.

## Figures and Tables

**Table 1:** Sensory evaluation of tea samples.

	F	W12	W20
Aroma description	Obvious grassy	Lower grassy with green odor	Green odor with moderate sweet and/or floral odor
Score	81	83	86

Note: F: fresh tea leaves; W12 and W20: tea leaves were withered for 12 h and 20 h, respectively.

**Table 2:** The contents (μg/L) and proportions (%) of different kinds of volatile compounds.

	F		W12		W20	
	Content	Proportion	Content	Proportion	Content	Proportion
Aromatic hydrocarbons	2396.38 ± 365.87 a	17.89	1331.43 ± 127.78 b	12.21	1044.59 ± 138.73 b	10.66
Alkanes	299.62 ± 28.16 a	2.23	129.24 ± 12.18 b	1.18	109.74 ± 7.18 b	1.12
Alkenes	3060.08 ± 526.11 a	22.72	1615.23 ± 90.77 b	14.82	1566.70 ± 122.58 b	16.01
Alcohols	3372.30 ± 766.58 a	24.89	3767.02 ± 302.55 a	34.56	4170.94 ± 107.80 a	42.71
Aldehydes	374.89 ± 35.21 a	2.80	621.91 ± 34.27 b	5.71	821.85 ± 80.45 c	8.41
Ketones	65.10 ± 1.76 a	0.49	67.85 ± 7.98 a	0.62	60.89 ± 3.50 a	0.62
Esters	3711.33 ± 301.17 a	27.89	3208.93 ± 192.90 b	29.46	1850.75 ± 107.55 c	18.93
Oxygen heterocyclic compounds	115.42 ± 17.63 a	0.86	148.26 ± 15.46 b	1.36	147.00 ± 9.34 b	1.50
Others	31.43 ± 3.22 a	0.23	7.82 ± 3.11 b	0.07	2.96 ± 0.35 c	0.03
Total	13,426.55 ± 1356.00 a	100.00	10,897.69 ± 621.15 b	100.00	9775.42 ± 328.71 b	100.00

Note: the data are shown as mean ± SD (n = 4); a, b, c: *p* < 0.05 for the changes with different letters (Tukey s-b (K) test); F: fresh tea leaves; W12 and W20: tea leaves were withered for 12 h and 20 h, respectively.

**Table 3:** The contents (μg/L) of volatile compounds of different origins.

		F	W12	W20
FADVs	Non-ester FADVs	372.64 ± 46.88 a	586.24 ± 53.70 b	717.77 ± 27.64 c
	Ester FADVs	2411.93 ± 119.32 a	2230.42 ± 93.91 b	971.80 ± 57.04 c
	Sum of FADVs	2784.57 ± 91.69 a	2816.66 ± 138.80 a	1689.56 ± 48.02 b
AADVs		1409.38 ± 211.14 a	1805.97 ± 183.07 b	1999.92 ± 109.06 b
VTs		4851.43 ± 975.20 a	4286.08 ± 404.06 a	4483.89 ± 92.72 a
CDVs		54.41 ± 2.46 a	69.88 ± 8.02 b	63.89 ± 3.13 b
Total		9099.79 ± 1133.05 a	8978.59 ± 500.67 a	8237.26 ± 157.94 a

Note: the data are shown as mean ± SD (n = 4); a, b, c: *p* < 0.05 for the changes with different letters (Tukey s-b (K) test); ester FADVs did not include the esters concurrently belonging to FADVs and AADVs; F: fresh tea leaves; W12 and W20: tea leaves were withered for 12 h and 20 h, respectively.

## Data Availability

The original contributions presented in this study are included in the article/Supplementary Materials. Further inquiries can be directed to the corresponding author.

## Supplementary Materials

The following supporting information can be downloaded at https://www.mdpi.com/article/10.3390/foods15111903/s1, Table S1: The moisture content of tea leaves during the withering period; Table S2: Identified volatile compounds and their contents during the withering period of Ningchow black tea.
