# Supplementary material for: Study on Aroma Formation During the Withering Period of Ningchow Black Tea
Source: Foods. 2026 May 28;15(11):1903. doi: 10.3390/foods15111903 (PMC13256084; doi:10.3390/foods15111903)
Supplement: Supplementary file 1 [file foods-15-01903-s001.zip › foods-4294839-supplementary.pdf]

Study on the aroma formation during the withering period of Ningchow black tea

Yingjie Huang [1,†], Ziyi Li [1,†], Yumei Ke [1], Juan Tu [2], Feng Xie [2], Caigang Yan [3], Kai Zhong [3], and Qincao Chen [1,*]

[1] College of Agriculture, Jiangxi Agricultural University, Nanchang 330045, China

[2] Horticulture Research Institute, Jiangxi Academy of Agricultural Sciences, Nanchang 330200, China

[3] Agriculture, Rural Affairs and Water Resources Bureau of Tonggu County, Yichun 336299, China

[†] These authors contributed equally to this work.

* **Corresponding Author**

Qincao Chen

chenqincao@jxau.edu.cn

Tel./fax: +86 571 86650617

Table S1 The moisture content of tea leaves during the withering period

Time	0 h	12 h	20 h
Moisture	77.30%	69.31%	61.57%

Note: F: fresh tea leaves; W12 and W20: tea leaves were withered for 12 h and 20 h, respectively. The moisture contents were determined using a rapid moisture meter (XY-110MW, Xingyun; Changzhou, China).

Table S2 Identified volatile compounds and their contents (μg/L) during the withering period of Ningchow black tea

NO.	Name	CAS	Tested RI	Referred RI	Category	Origin	F	W12	W20	*P*
1	Toluene	108-88-3	759.2	763	Aromatic hydrocarbons		140.82±54.02	83.82±10.2	80.21±4.25	0.043
2	Ethylbenzene	100-41-4	855.7	866	Aromatic hydrocarbons		158.93±58.09	98.53±8.51	79.95±8.6	0.024
3	1,3-Dimethylbenzene	108-38-3	864.5	866	Aromatic hydrocarbons		255.18±74.63	160.28±18.18	121.76±15.37	0.007
4	Styrene	100-42-5	886.8	893	Aromatic hydrocarbons		1092.1±150.7	799.74±93.26	594.4±100.45	0.001
5	(1-Methylethyl)benzene	98-82-8	921.7	921	Aromatic hydrocarbons		4.51±0.92	4.11±0.34	3.72±0.23	0.211
6	Propylbenzene	103-65-1	950.5	953	Aromatic hydrocarbons		5.49±0.63	3.46±0.59	2.5±0.59	0.000
7	o-Cymene	527-84-4	1022.9	1022	Aromatic hydrocarbons		219.58±30.63	139.56±6.48	136.37±9.49	0.000
8	Naphthalene	91-20-3	1176.0	1182	Aromatic hydrocarbons		519.77±61.82	41.93±4.4	25.68±0.81	0.000
9	Octane	111-65-9	800.5	800	Alkanes		1.91±0.6	3.84±0.46	5.67±0.22	0.000
10	Nonane	111-84-2	900.1	900	Alkanes		0.83±0.16	0.4±0.11	0.34±0.06	0.000
11	Decane	124-18-5	1001.0	1000	Alkanes		16.59±3.13	3.3±0.4	2.27±0.19	0.000
12	5-Methyldecane	13151-35-4	1057.7	1057	Alkanes		5.34±0.97	4.78±0.13	5.73±0.12	0.114
13	2-Methyldecane	6975-98-0	1064.2	1064	Alkanes		28.31±3.84	12.28±1.22	7.81±1.13	0.000
14	3-Methyldecane	13151-34-3	1070.8	1071	Alkanes		47.64±5.08	35.88±3.48	33.87±0.65	0.001
15	Undecane	1120-21-4	1100.0	1100	Alkanes		139.51±25.32	31.53±5.01	27.62±2.32	0.000
16	5-Methylundecane	1632-70-8	1156.3	1156	Alkanes		1.82±0.33	1.45±0.15	0.98±0.36	0.010
17	3-Methylundecane	1002-43-3	1170.6	1170	Alkanes		17.47±4.35	11.71±0.98	10.16±0.46	0.007
18	Tridecane	629-50-5	1299.9	1300	Alkanes		29.59±11.16	17.52±2.66	11.49±2.08	0.012
19	5-Methyltridecane	25117-31-1	1354.1	1348	Alkanes		1.37±0.57	0.58±0.06	0.39±0.1	0.006
20	3-Methyltridecane	6418-41-3	1370.5	1371	Alkanes		7.71±4.77	5.01±0.6	2.86±0.58	0.099
21	Hexadecane	544-76-3	1599.7	1600	Alkanes		1.55±0.94	0.97±0.15	0.57±0.11	0.091
22	4-Methyl-1,3-pentadiene	926-56-7	642.7	629	Alkenes		0.73±0.61	0.39±0.22	0.51±0.45	0.592
23	1-Nonene	124-11-8	890.9	889	Alkenes		0.96±0.23	0.17±0.04	0.16±0.03	0.000
24	Camphene	79-92-5	943.4	952	Alkenes		1.93±0.52	2.12±0.31	2.16±0.22	0.669
25	3-Ethyl-1,5-octadiene		944.5	947	Alkenes		nd	0.28±0.02	0.38±0.06	0.020
26	β-Myrcene	123-35-3	992.0	991	Alkenes	VT	943.04±135.86	682.66±31.26	682.09±41.92	0.002

27	Terpilene	99-86-5	1016.5	1017	Alkenes	VT	29.12±8	30.36±3.5	33.52±1.84	0.489
28	Limonene	138-86-3	1028.1	1030	Alkenes	VT	917.97±134.73	391.2±42.1	373.21±50.98	0.000
29	β-Ocimene	13877-91-3	1038.9	1037	Alkenes	VT	121.64±37.92	137.22±18.55	150.31±6.91	0.307
30	trans-β-Ocimene	3779-61-1	1048.6	1049	Alkenes	VT	255.82±80.76	181.05±21.9	179.9±12.83	0.091
31	γ-Terpinene	99-85-4	1056.6	1060	Alkenes	VT	26.2±6.28	25.93±2.65	26.71±1.56	0.962
32	(E)-4,8-Dimethylnona-1,3,7-triene	19945-61-0	1118.1	1116	Alkenes		597.11±139.53	31.73±6.5	8.04±1.41	0.000
33	Cosmene		1121.6	1131	Alkenes	VT	10.02±2.23	6.77±0.57	7.02±0.52	0.014
34	(E,E)-Cosmene	460-01-5	1129.3	1131	Alkenes	VT	34.73±7.54	23.18±2.22	24.01±1.56	0.011
35	(4E,6E)-Alloocimene	3016-19-1	1140.8	1144	Alkenes	VT	54.85±11.26	38.88±1.21	36.71±2.92	0.008
36	α-Cubebene	17699-14-8	1347.6	1351	Alkenes	VT	2.24±0.4	2.35±0.42	1.65±0.12	0.039
37	Copaene	3856-25-5	1373.0	1376	Alkenes	VT	4.15±0.63	5.2±0.86	3.66±0.32	0.022
38	β-Bourbonene	5208-59-3	1381.6	1384	Alkenes	VT	6.45±0.52	4.92±1.12	3.22±0.68	0.001
39	β-Caryophyllene	87-44-5	1415.7	1419	Alkenes	VT	1.92±0.36	1.91±0.33	1.33±0.18	0.035
40	(E)-β-Famesene	18794-84-8	1457.1	1457	Alkenes	VT	4.28±1.55	2.25±0.91	1.43±0.15	0.010
41	γ-Muurolene	30021-74-0	1474.2	1477	Alkenes	VT	1.15±0.2	1.1±0.22	0.67±0.03	0.006
42	α-Curcumene	644-30-4	1481.8	1483	Alkenes	VT	1.25±0.42	0.54±0.13	0.27±0.03	0.001
43	α-Muurolene	10208-80-7	1497.5	1499	Alkenes	VT	3.78±0.83	3.97±0.69	2.79±0.14	0.054
44	α-Farnesene	502-61-4	1507.9	1508	Alkenes	CDV	1.81±0.55	2.06±0.5	0.74±0.07	0.004
45	cis-Calamenene	483-77-2	1520.7	1523	Alkenes	VT	32.05±8.45	31.41±4.05	20.9±0.59	0.030
46	δ-Cadinene	483-76-1	1521.3	1524	Alkenes	VT	5.7±0.96	6.8±1.56	5.07±0.24	0.121
47	4,8,12-trimethyltrideca-1,3,7,11-tetraene	62235-06-7	1579.2	1577	Alkenes	VT	1.18±0.22	0.77±0.29	0.24±0.04	0.001
48	(Z)-2-Penten-1-ol	1576-95-0	765.2	767	Alcohols		0.46±0.15	3.47±1.32	2.33±0.72	0.003
49	(Z)-3-Hexen-1-ol	928-96-1	854.7	857	Alcohols	FADV	19.55±8.86	63.94±11.54	60.55±3.44	0.000
50	(E)-2-Hexenol	928-95-0	868.4	862	Alcohols	FADV	2.55±1.27	86.48±14.09	132.37±6.02	0.000
51	1-Hexanol	111-27-3	869.8	868	Alcohols	FADV	17.34±5.48	120.11±13.11	211.98±11.39	0.000
52	1-Heptanol	111-70-6	974.3	970	Alcohols	FADV	8.85±2.58	13.4±1.64	21.63±1.54	0.000
53	1-Octen-3-ol	3391-86-4	983.0	980	Alcohols	FADV	28.82±7.44	55.43±7.29	63.1±4.61	0.000
54	6-Methyl-5-hepten-2-ol	1569-60-4	995.4	994	Alcohols	CDV	2.52±0.89	6.14±0.8	8.37±0.5	0.000

55	Anhydrolinalool oxide	54750-69-5	1006.9	1008	Alcohols	VT	nd	1.7±0.42	2.6±0.4	0.021
56	Benzyl alcohol	100-51-6	1033.3	1036	Alcohols	AADV	389.6±63.19	167.52±16.96	171.87±20.57	0.000
57	cis-Linalool oxide (furanoid)	5989-33-3	1071.6	1074	Alcohols	VT	111.12±36.12	160.6±19.42	173.12±10.62	0.014
58	trans-Linalool oxide (furanoid)	34995-77-2	1087.1	1086	Alcohols	VT	182.45±62.56	246.63±37.93	261.26±17.9	0.067
59	Linalool	78-70-6	1105.2	1099	Alcohols	VT	1732.06±432.13	1884.63±213.06	2010.02±17.09	0.405
60	Hotrienol	29957-43-5	1107.5	1107	Alcohols	VT	16.37±3.74	9.25±4.02	6.53±2.22	0.008
61	Phenylethyl Alcohol	60-12-8	1111.7	1116	Alcohols	AADV	501.58±110.69	540.57±151.01	557.99±67.85	0.782
62	(Z)-3-Nonen-1-ol	10340-23-5	1155.3	1156	Alcohols	FADV	1.19±0.34	4.27±0.76	8.93±0.54	0.000
63	(E,Z)-3,6-Nonadienol	56805-23-3	1158.4	1156	Alcohols	FADV	0.69±0.12	3.9±0.76	8.82±0.68	0.000
64	cis-Linalool oxide (pyranoid)	14009-71-3	1168.1	1173	Alcohols	VT	36.02±11.03	43.84±13.88	38.44±5.23	0.588
65	1-Nonanol	143-08-8	1173.5	1173	Alcohols	FADV	13.71±2.97	11.15±1.5	15.75±1.28	0.034
66	trans-Linalool oxide (pyranoid)	39028-58-5	1173.4	1173	Alcohols	VT	28.43±8.58	35.52±11.89	31±4.52	0.542
67	Terpinen-4-ol	20126-76-5	1175.0	1182	Alcohols	VT	3.08±0.42	3.76±0.38	3.31±0.26	0.066
68	α-Terpineol	98-55-5	1188.8	1189	Alcohols	VT	2.23±0.59	2.5±0.36	2.87±0.16	0.142
69	Nerol	106-25-2	1228.3	1228	Alcohols	VT	13.78±5.33	18.31±3.85	22.74±1.48	0.030
70	trans-Isogeraniol		1233.9	1240	Alcohols	VT	2.6±1.02	2.94±1.03	4.57±0.54	0.027
71	cis-Isogeraniol	5944-20-7	1245.0	1240	Alcohols	VT	0.24±0.05	0.94±0.52	1.16±0.17	0.007
72	Geraniol	106-24-1	1256.9	1255	Alcohols	VT	245.97±133.98	275.92±48.35	347.05±30.6	0.269
73	1-Decanol	112-30-1	1273.7	1273	Alcohols	FADV	0.41±0.12	0.25±0.05	0.41±0.07	0.040
74	Nerolidol	7212-44-4	1563.6	1564	Alcohols	VT	9.85±3.29	2.82±1.63	1.48±0.03	0.001
75	δ-Cadinol	19435-97-3	1641.1	1645	Alcohols	VT	0.82±0.32	1.02±0.23	0.71±0.05	0.216
76	2-Methyl-propanal	78-84-2			Aldehydes	AADV	nd	0.07±0.02	0.05±0.03	0.373
77	3-Methyl-butanal	590-86-3	655.8	652	Aldehydes	AADV	0.27±0.19	0.43±0.41	1.8±1.1	0.022
78	2-Methyl-butanal	96-17-3	663.5	662	Aldehydes	AADV	1.03±0.73	8.55±4.19	23.75±17.72	0.037
79	Hexanal	66-25-1	801.9	800	Aldehydes	FADV	4.4±1.21	21.03±5.07	25.45±1.73	0.000
80	(Z)-2-Hexanal		845.9	854	Aldehydes	FADV	nd	0.25±0.03	0.45±0.07	0.002
81	(E)-2-Hexanal	6728-26-3	852.2	854	Aldehydes	FADV	1.61±1.05	28.96±4.78	37.91±2.16	0.000
82	Heptanal	111-71-7	902.1	901	Aldehydes	FADV	12.97±3.67	10.86±2.08	17.21±0.95	0.016

83	Benzaldehyde	100-52-7	956.4	962	Aldehydes	AADV	78.48±12.19	243.98±32.94	291.39±26.67	0.000
84	(E)-2-Heptenal	18829-55-5	956.8	958	Aldehydes	FADV	0.55±0.18	1.57±0.15	2.25±0.26	0.000
85	(E,Z)-2,4-Heptadienal	4313-02-4	996.9	998	Aldehydes	FADV	1.33±0.16	2.29±0.36	3.42±0.33	0.000
86	Octanal	124-13-0	1003.5	1003	Aldehydes	FADV	6.54±1.06	5.57±0.31	6.6±0.61	0.133
87	(E,E)-2,4-Heptadienal	4313-03-5	1010.1	1012	Aldehydes	FADV	202.63±23.73	113.46±3.38	41.03±0.84	0.000
88	Benzeneacetaldehyde	122-78-1	1042.1	1045	Aldehydes	AADV	7.55±1.65	121.9±7.28	286.29±63.93	0.000
89	(E)-2-Octenal	2548-87-0	1058.0	1060	Aldehydes	FADV	8.8±3.27	5.27±0.64	6.16±0.62	0.074
90	Nonanal	124-19-6	1107.3	1104	Aldehydes	FADV	26.78±6.81	20.51±12.3	34.46±1.66	0.105
91	α-Cyclocitral	432-24-6	1114.4	1116	Aldehydes	CDV	nd	1.05±0.13	0.63±0.06	0.001
92	(E,E)-2,6-Nonadienal	17587-33-6	1153.3	1152	Aldehydes	FADV	1.08±0.23	6.32±0.63	8.39±0.68	0.000
93	(E)-2-Nonenal	18829-56-6	1159.9	1162	Aldehydes	FADV	1.37±0.28	2.84±0.23	2.09±0.22	0.000
94	Safranal	116-26-7	1197.1	1201	Aldehydes	CDV	0.34±0.05	0.69±0.06	0.69±0.04	0.000
95	Decanal	112-31-2	1205.8	1206	Aldehydes	FADV	8.58±3.14	6.4±0.63	6.88±0.4	0.270
96	β-Cyclocitral	432-25-7	1217.9	1220	Aldehydes	CDV	0.48±0.09	0.73±0.04	0.76±0.02	0.000
97	(Z)-Neral	106-26-3	1241.0	1240	Aldehydes	VT	0.97±0.84	3.91±0.45	4.04±0.66	0.000
98	β-Cyclohomocitral	472-66-2	1255.0	1254	Aldehydes	CDV	0.59±0.13	0.71±0.03	0.63±0.18	0.478
99	(E)-2-Decenal	3913-81-3	1262.0	1263	Aldehydes	FADV	0.52±0.12	1.03±0.1	1.19±0.16	0.000
100	(E)-Geranial	141-27-5	1271.0	1270	Aldehydes	VT	7.75±3.8	13.08±2.1	17.85±1.35	0.001
101	2-Phenyl-2-butenal	4411-89-6	1271.4	1279	Aldehydes		0.27±0.13	0.44±0.07	0.45±0.05	0.036
102	2-Heptanone	110-43-0	893.8	891	Ketones		0.52±0.1	0.43±0.05	0.7±0.05	0.001
103	1-Octene-3-one	4312-99-6	985.6	979	Ketones	FADV	2.37±0.25	0.94±0.18	0.72±0.06	0.000
104	6-Methyl-5-hepten-2-one	110-93-0	988.9	986	Ketones	CDV	2.45±0.29	3.57±0.32	4.69±0.94	0.002
105	2,2,6-Trimethylcyclohexanone	2408-37-9	1031.4	1036	Ketones	CDV	35.76±3.74	36.87±5.72	36.63±3.1	0.930
106	Acetophenone	98-86-2	1064.2	1065	Ketones		8.59±2.66	5.44±0.56	3.84±0.44	0.007
107	3-(Hydroxymethyl)-2-nonanone	67801-33-6	1093.7	1093	Ketones		5.22±0.41	1.81±0.46	1.95±0.23	0.000
108	(R,S)-5-Ethyl-6-methyl-3E-hepten-2-one	57283-79-1	1147.4	1144	Ketones		0.59±0.05	1.53±0.26	2.39±0.13	0.000
109	(E)-β-Damascenone	23726-93-4	1383.3	1386	Ketones	CDV	6.95±0.61	10.13±0.78	4.48±0.24	0.000
110	α-Ionone	127-41-3	1426.3	1426	Ketones	CDV	0.23±0.05	0.58±0.13	0.51±0.03	0.000

111	Geranyl acetone	3796-70-1	1453.3	1453	Ketones	CDV	2.27±1.64	6.02±0.76	4.5±0.18	0.002
112	β-Ionone	14901-07-6	1484.6	1491	Ketones	CDV	0.16±0.02	0.52±0.08	0.47±0.02	0.000
113	Methyl butyrate	623-42-7	723.0	722	Esters		2.13±0.67	0.93±0.15	1.02±0.23	0.005
114	Ethyl butanoate	105-54-4	803.5	802	Esters		28.8±7.01	9.71±1.67	6.31±1.85	0.000
115	Butyl acetate	123-86-4	816.7	812	Esters		18.01±4.35	6.96±1.19	5.44±1.69	0.000
116	Ethyl (E)-2-butenoate	623-70-1	845.4	835	Esters		10.14±2.77	4.49±0.84	4.17±1.63	0.003
117	3-Methyl-1-butyl acetate	123-92-2	879.4	876	Esters	AADV	1.17±0.31	0.34±0.06	0.4±0.03	0.000
118	Propyl butanoate	105-66-8	901.6	896	Esters		4.78±1.07	1.64±0.32	1.39±0.49	0.000
119	Ethyl pentanoate	539-82-2	905.1	900	Esters		0.88±0.19	0.21±0.09	0.44±0.09	0.000
120	Butyl propanoate	590-01-2	912.8	908	Esters		0.93±0.22	0.4±0.06	0.34±0.1	0.001
121	3-Methyl-2-buten-1-yl acetate	1191-16-8	917.2	918	Esters		1.04±0.35	0.69±0.08	0.42±0.04	0.007
122	(Z)-3-Hexenyl formate	33467-73-1	922.4	920	Esters	FADV	0.94±0.29	1.16±0.14	1±0.07	0.287
123	Methyl hexanoate	106-70-7	927.2	925	Esters	FADV	27.64±8.87	10.91±1.73	9.83±3.17	0.002
124	Methyl 3-hexenoate	2396-78-3	935.9	933	Esters	FADV	0.14±0.07	0.49±0.23	0.23±0.04	0.015
125	Butyl butanoate	109-21-7	998.5	995	Esters		790.29±217.86	229.31±53.68	187.76±79.53	0.000
126	Ethyl hexanoate	123-66-0	1002.2	1000	Esters	FADV	218.24±66.08	59.21±15.23	34.75±16.47	0.000
127	(Z)-3-Hexenyl acetate	3681-71-8	1009.9	1005	Esters	FADV	1426.37±161.28	780.59±25.97	229.73±17.17	0.000
128	Hexyl acetate	142-92-7	1017.0	1011	Esters	FADV	61.12±15.53	61.31±5.55	41.3±4.18	0.028
129	(Z)-2-Hexenyl acetate	56922-75-9	1020.0	1006	Esters	FADV	24.63±2.43	77.55±7.23	32.79±0.68	0.000
130	Butyl 2-butenoate	7299-91-4	1045.5	1046	Esters		42.14±24.29	7.71±2.32	6.25±3.4	0.010
131	Ethyl 2-hexenoate	1552-67-6	1046.8	1037	Esters	FADV	12.08±6.57	2.46±0.7	1.89±0.98	0.008
132	(E)-2-Hexenyl propanoate	53398-80-4	1114.1	1111	Esters	FADV	6.73±1.43	20.59±2.2	12.53±0.51	0.000
133	Methyl octanoate	111-11-5	1127.2	1126	Esters	FADV	11.72±11.22	2.27±2.04	0.84±0.21	0.088
134	cis-3-Hexenyl iso-butyrate	41519-23-7	1145.9	1145	Esters	FADV/AADV	1.79±0.19	9.69±0.56	10.2±1.21	0.000
135	Hexyl isobutyrate	2349-07-7	1151.2	1150	Esters	FADV/AADV	nd	0.52±0.04	1.82±0.26	0.000
136	(E)-3-Hexenyl butanoate	53398-84-8	1188.4	1185	Esters	FADV	186.95±13.41	448.31±20.88	176.2±7.11	0.000
137	Methyl salicylate	119-36-8	1191.8	1192	Esters	AADV	353.22±87.8	552.69±123.64	492.89±64.22	0.041
138	Butyl hexanoate	626-82-4	1192.9	1189	Esters	FADV	92.88±85.01	9.06±3.45	6.54±2.41	0.057

139	Hexyl butyrate	2639-63-6	1193.9	1192	Esters	FADV	97.81±83.64	33.32±4.45	35.1±3.8	0.156
140	(E)-2-Hexenyl butyrate	53398-83-7	1197.3	1195	Esters	FADV	22.78±1.73	316.13±16.87	186.18±5.94	0.000
141	cis-3-Hexenyl-α-methylbutyrate	53398-85-9	1233.8	1234	Esters	FADV/AADV	17.23±1.97	59.44±2.67	43.64±3.01	0.000
142	cis-3-Hexenyl isovalerate	35154-45-1	1237.7	1238	Esters	FADV/AADV	21.19±1.83	50.95±3.11	61.85±3.83	0.000
143	Hexyl 2-methylbutyrate	10032-15-2	1238.4	1236	Esters	FADV/AADV	0.88±0.14	6.12±0.31	12.52±0.9	0.000
144	cis-2-Hexenyl isovalerate		1240.4	1245	Esters	FADV/AADV	1.52±0.15	16.39±0.6	15.18±1.16	0.000
145	Butyl (E)-2-hexenoate	54411-16-4	1242.5	1243	Esters	FADV	3.29±3.41	0.48±0.1	0.48±0.06	0.119
146	trans-2-Hexenyl isovalerate	68698-59-9	1246.5	1245	Esters	FADV/AADV	0.62±0.19	15.75±1.11	23.15±1.15	0.000
147	3-Methylbutyl hexanoate	2198-61-0	1251.7	1252	Esters	FADV/AADV	0.76±0.5	0.56±0.1	0.67±0.04	0.637
148	trans-2-Hexenyl valerate	56922-74-8	1285.6	1299	Esters	FADV/AADV	1.23±0.12	3.24±0.34	1.74±0.04	0.000
149	Methyl geranoate	1189-09-9	1324.8	1324	Esters	VT	0.13±0.04	0.23±0.07	0.45±0.12	0.001
150	(E)-Hex-3-enyl (E)-2-methylbut-2-enoate		1325.2	1319	Esters	FADV	0.79±0.18	4.46±1.01	7.3±2.55	0.001
151	cis-3-Hexenyl hexanoate	31501-11-8	1382.6	1380	Esters	FADV	207.73±6.82	330.83±27.5	138.89±6.77	0.000
152	cis-3-Hexenyl cis-3-hexenoate	61444-38-0	1387.6	1389	Esters	FADV	4.58±1.72	15.53±1.04	21.43±0.34	0.000
153	(E)-2-Hexenyl hexanoate	53398-86-0	1391.1	1391	Esters	FADV	5.53±0.66	55.76±4.52	34.79±0.55	0.000
154	Dihydroactinidiolide	17092-92-1	1523.3	1532	Esters	CDV	0.18±0.04	0.18±0.06	0.13±0.03	0.312
155	cis-3-Hexenyl benzoate	25152-85-6	1569.7	1570	Esters	FADV/AADV	0.35±0.05	0.35±0.05	0.79±0.16	0.000
156	2-Ethyl-furan	3208-16-0	706.0	703	Oxygen heterocyclic compounds		1.33±0.52	8.1±1.92	7.26±2.13	0.001
157	2-Butylfuran	4466-24-4	900.0	893	Oxygen heterocyclic compounds		0.95±0.28	3.49±0.48	4.81±0.28	0.000
158	2,6,6-Trimethyl-2-ethenyltetrahydropyran	7392-19-0	970.1	972	Oxygen heterocyclic compounds		0.9±0.22	1.03±0.12	1.1±0.05	0.211
159	2-Pentylfuran	3777-69-3	991.5	993	Oxygen heterocyclic compounds		35.17±6.39	87.47±11.61	83.81±6.8	0.000
160	cis-2-(2-Pentenyl)furan	70424-13-4	1001.7	1002	Oxygen heterocyclic compounds		76.37±12.12	47.53±2.7	49.39±4.04	0.001
161	cis-theaspirane	36431-72-8	1294.9	1302	Oxygen heterocyclic compounds	CDV	0.35±0.09	0.31±0.15	0.28±0.04	0.658
162	(E)-Theaspirane		1312.3	1303	Oxygen heterocyclic compounds	CDV	0.36±0.03	0.33±0.02	0.36±0.04	0.358
163	Dimethyl sulfide	75-18-3			Others	AADV	0.05±0.02	0.23±0.15	0.24±0.02	0.027
164	Benzyl nitrile	140-29-4	1138.3	1144	Others	AADV	30.86±3.28	6.65±3.15	1.69±0.36	0.000
165	Indole	120-72-9	1290.3	1295	Others		0.52±0.17	0.94±0.24	1.04±0.03	0.004

Note: The data are shown as mean ± SD (n=4); nd: the compound was not detected; *P*: the difference significances were calculated by ANOVA; F: fresh tea leaves; W12 and W20: tea leaves were withered for 12 h and 20 h, respectively.
